# Decreased Thalamocortical Functional Connectivity after 36 Hours of Total Sleep Deprivation: Evidence from Resting State fMRI

**DOI:** 10.1371/journal.pone.0078830

**Published:** 2013-10-25

**Authors:** Yongcong Shao, Lubin Wang, Enmao Ye, Xiao Jin, Wei Ni, Yue Yang, Bo Wen, Dewen Hu, Zheng Yang

**Affiliations:** 1 Beijing Institute of Basic Medical Sciences, Beijing, PR China; 2 Cognitive and Mental Health Research Center, Beijing, PR China; 3 College of Mechatronics and Automation, National University of Defense Technology, Changsha, Hunan, PR China; 4 Department of Radiology, The General Hospital of the People's Liberation Army, Beijing, PR China; University of Pennsylvania, United States of America

## Abstract

**Objectives:**

The thalamus and cerebral cortex are connected via topographically organized, reciprocal connections, which hold a key function in segregating internally and externally directed awareness information. Previous task-related studies have revealed altered activities of the thalamus after total sleep deprivation (TSD). However, it is still unclear how TSD impacts on the communication between the thalamus and cerebral cortex. In this study, we examined changes of thalamocortical functional connectivity after 36 hours of total sleep deprivation by using resting state function MRI (fMRI).

**Materials and Methods:**

Fourteen healthy volunteers were recruited and performed fMRI scans before and after 36 hours of TSD. Seed-based functional connectivity analysis was employed and differences of thalamocortical functional connectivity were tested between the rested wakefulness (RW) and TSD conditions.

**Results:**

We found that the right thalamus showed decreased functional connectivity with the right parahippocampal gyrus, right middle temporal gyrus and right superior frontal gyrus in the resting brain after TSD when compared with that after normal sleep. As to the left thalamus, decreased connectivity was found with the right medial frontal gyrus, bilateral middle temporal gyri and left superior frontal gyrus.

**Conclusion:**

These findings suggest disruptive changes of the thalamocortical functional connectivity after TSD, which may lead to the decline of the arousal level and information integration, and subsequently, influence the human cognitive functions.

## Introduction

Sleep deprivation (SD) can potentially lead to deficits in many cognitive and affective capacities [1], especially for sustained or vigilant attention which are particularly robust and are of great importance in predicting real-world cognitive errors [2]. These negative impacts on cognition and affective process induced by SD in human strongly suggested that sleep pressure represented a basic physiological constraint of brain function [3]–[7]. Recently, task-dependent fMRI studies have identified the brain attention and control network areas such as the thalamus, basal ganglia, cerebellum, frontal and parietal cortexes are associated with these cognitive deficits, indicating the brain functions are adversely affected after SD [7]–[10]. However, the neural mechanism underlying the decline awareness and cognition induced by SD is far from clear.

The thalamus is a vital region which seems to integrate neural activity from widespread neocortical inputs and outputs [11]. Thalamic nuclei have strong reciprocal connections with the cerebral cortex, forming thalamo-cortico-thalamic circuits that are involved with consciousness. The thalamus is believed to modulate and facilitate communication in all areas of the cerebral cortex, and plays an important role in regulating state of sleep and wakefulness [12]. In the past years, neuroimaging studies including PET and functional MRI (fMRI) have investigated the thalamic activity after TSD, which greatly advanced our understanding of the neurophysiological mechanism of sleep deprivation. By using working memory paradigms, fMRI studies have reported decreases in the activation of the thalamus and the parietal cortex after TSD [8]–[10]. These results are consistent with those positron emission tomography (PET) studies which indicated that cerebral metabolism in the thalamus, basal ganglia, cerebellum, frontal and parietal cortex was decreased following SD [13]. Decreased performance was positively correlated with decreased metabolic rate for several prefrontal cortex regions and for the thalamus, supporting the vulnerability of the thalamocortical circuits that are so critical for higher order cognitive functions [9]. Besides, Tomasi and his colleagues found that task difficulty was associated with an inverse relationship between parietal cortex and thalamus activity (decreased activity in the parietal cortex and increased activity in the thalamus) [7]. This suggests that thalamic hyperactivation during SD could underlie the reduced activation in the parietal lobe and the blunted deactivation in cingulate cortices, which represents the impaired attention networks after TSD.

Recently, resting-state fMRI has attracted increasing attention and been used in investigating various brain functions. It examines temporal correlations in intrinsic low-frequency fluctuations in the blood-oxygen-level dependent (BOLD) signal between brain regions, which sidesteps many of the limitations associated with conventional task-based functional imaging and has proven useful for mapping brain networks [14]–[16]. Several reports described the changes in functional connectivity in the brain after partial and short-time SD [4], [17]. Recent evidences suggest that even one night of controlled SD in the laboratory is associated with reduced functional connectivity within the default mode network (DMN) and anti-correlated network (ACN) [4]. Similarly, restriction of sleep to 3.5 hours during a single night in the laboratory was also associated with reduced functional connectivity of DMN and ACN nodes, suggesting that SD induces a robust alteration in the intrinsic connectivity within and between these networks [17].

Taken together, most studies have focused on the DMN and ACN functional connectivity regarding their crucial roles in segregating internally and externally directed awareness, while little attention has been devoted to the potential role of subcortical nuclei such as thalamus. As the thalamus has a key role in regulating oscillatory brain activity in state of sleep and wakefulness [12], it is important to examine how TSD impact on the connectivity of thalamus. In this study, we hypothesized that resting-state thalamus connectivity with other brain regions would be disrupted after TSD. By selecting thalamus as “seed voxel” in the well-restated state, we tested our hypothesis by comparing whole-brain functional connectivity pattern before and after 36 hours of TSD, and expect to add new evidence that sleep deprivation altered resting brain function.

## Materials and Methods

### Subjects

Sixteen subjects were recruited from Beijing Normal University as paid volunteers by advertisement. Two subjects did not fulfill the entire experiment for some reasons. The remaining fourteen subjects were included in this study (age: range 18–28; Mean±S.D. 25.9±2.3 years old). All subjects were right-handed with normal or corrected normal vision. None of them had previously participated in psycho-physiological experiments. The general exclusion criteria included diseases of the central and peripheral nervous systems, encephalic traumatisms, cardiovascular diseases and/or hypertension, cataracts and/or glaucoma, pulmonary problems, audio logical problems, and alcohol and drug abuse. All participants had normal intelligence scores (the Raven test, IQ>100) and had no evidence of clinical symptom levels as assessed by the Symptom Checklist-90 (SCL-90) (T-scores were <60 on the General Symptom Index; the clinical range for the general population involves T scores >63). All subjects were required to maintain a regular sleep schedule and refrain from alcohol, caffeine, and chocolate intake and napping for 1 week before the study and for the duration of the study itself. The experimental protocol was approved by the Beijing Institute of Basic Medical Science and the Fourth Military Medical University (Xi’an, China). All subjects were given written informed consent after the experiment had been fully explained and then established a typical sleep pattern, defined as 8 h of sleep.

### Imaging Methods

**Figure 1:**
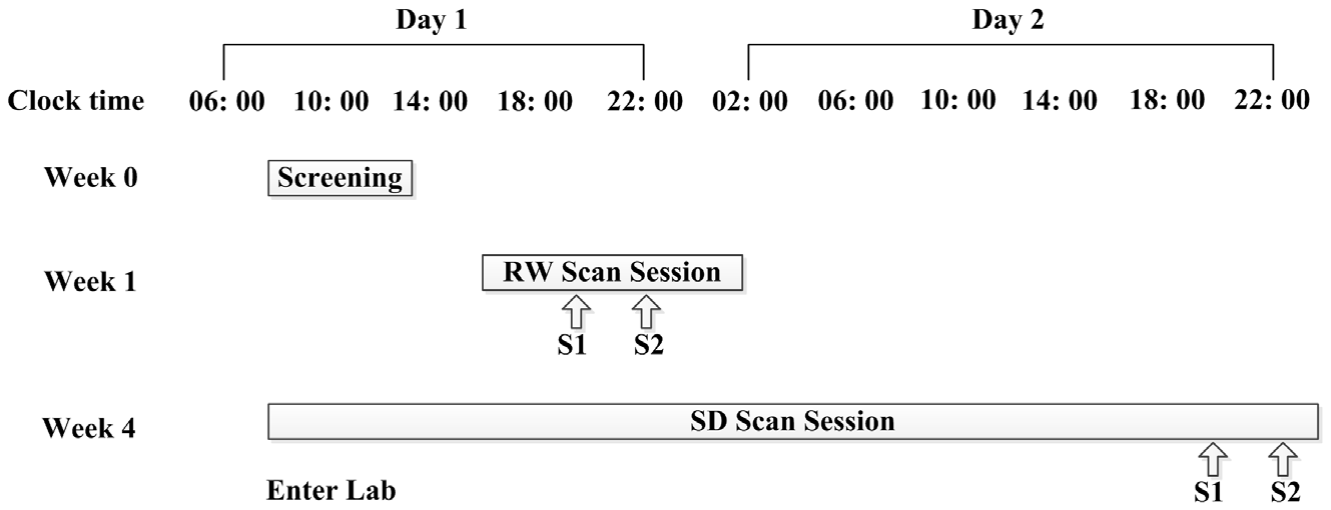
Study design schematic showing the three phases of the study and the times of day volunteers (S1 and S2) underwent briefing and brain imaging, respectively. The order of scanning was counterbalanced across subjects.

The MRI scans were conducted using a GE 3.0T Signa scanner with a birdcage RF head coil in the General Hospital of the PLA of China. Subjects were scanned twice, once during rested wakefulness (RW) and once after 36 h of TSD. The two scanning sessions were conducted 3 week apart to minimize the possibility of residual effects of SD affecting cognition of volunteers who underwent a SD scan before a RW scan. The order of scanning was counterbalanced across subjects to reduce the potential influence of order effects. Both of the scan sessions were performed at the same time (8:00 pm). The experiment was carried out in the basic aerospace institute with nursing staff present at all times. Each subject always had a partner in the study to help keeping each other awake through the night while under continuous behavioral monitoring. Subjects were not allowed to leave the Lab during the TSD period until they were escorted to the fMRI facility. A schematic describing our experimental design is shown in Fig. 1.

The scanning sessions included: (i) localization, (ii) T1-flair anatomy, (iii) one resting-state session, followed by several task-state fMRI sessions (Go/Nogo, working memory), (iv) high-resolution spoiled gradient recalled echo (SPGR) anatomy. Task-state data were not presented in the current study. During the resting-state scans, subjects were instructed simply to keep their eyes closed, be as motionless as possible and not to think of anything in particular. The scan lasted for 396 s. Foam padding was used to limit head movements within the coil. Earplugs were used to attenuate scanner noise. Functional images were obtained by a single-shot EPI sequence (TR: 2 s, TE: 30 ms, FOV: 256 mm, 3.75×3.75 mm in-plane resolution) of twenty axial slices (6-mm thick) covering the entire brain and measuring the BOLD signals. To make sure the subjects did not fall asleep during the scan, subjects were reminded to keep awake through microphone before each session. After each session, subjects were asked whether they were awake in the previous session and all the subjects confirmed that they were awake.

Subjects' heart and respiration rates were continuously monitored and recorded throughout the session using an MR-compatible pulse oximeter and respiratory bellows. The MR-compatible pulse oximeter was attached to the middle finger of the left hand and the respiratory bellows were strapped around the lower rib cage.

### Data Preprocessing

For the resting-state fMRI data, prior to preprocessing, the first 10 volumes of each scan were discarded to remove possible T1 stabilization effects. All resting-state images were preprocessed using the statistical parametric mapping software package (SPM8, Wellcome Department of Cognitive Neurology, Institute of Neurology, London, UK). The data were realigned to the first volume to correct for inter-scan head motions. All subjects in this study had less than 2 mm translation and 2° of rotation in any of the x, y, and z axes. Then, the volumes were normalized to the standard EPI template in the Montreal Neurological Institute (MNI) space and resliced to 3×3×3 mm^3^. The resulting images were spatially smoothed with a Gaussian filter of 6 mm full-width half-maximum kernel. The smoothed images were temporally band-pass filtered (0.01-0.1 Hz), followed by linear detrending to remove any residual drift. Nine nuisance signals were removed from the time series of each voxel via linear regression, including white matter (WM) signal, cerebrospinal fluid (CSF) signal, the whole-brain signal, and six motion parameters. The whole-brain signal was generated by averaging across the times series of all voxels in the brain. The WM and CSF signals were generated by averaging across the times series of a region centered in the white matter and a ventricular region of interest, separately. The six motion parameters were obtained by rigid body correction of head motion. This regression procedure was utilized to reduce spurious variance unlikely to reflect neural activity.

### Group Analyses

The left thalamus and right thalamus were defined as the seed regions for functional connectivity analysis. The masks of the seed regions were created in the MNI space using the software WFU_PickAtlas (http://www.ansir.wfubmc.edu). For each seed region and for each scan, the correlation map was created by calculating Pearson's correlation coefficients between the seed time series and time series of all voxels in the brain. A Fisher's r-to-z transformation was applied to improve the normality of these correlation coefficients. Group analyses were performed for the correlation maps of each seed region. First, correlation maps of subjects before and after the 36 h TSD were separately underwent two-tailed one-sample *t*-test, to determine brain regions with significant positive or negative correlations to the thalamus. Then, two-tailed paired *t*-test was conducted between the correlation maps of the RW and TSD, to identify significant TSD-related functional connectivity changes. The resulting statistical maps were set at a combined threshold of *p*<0.001 at the voxel level (uncorrected) with *p*<0.05 at the cluster level (FDR corrected). For the maps which did not meet this criterion, a relaxed threshold of *p*<0.001 at the voxel level (uncorrected) with cluster size > 20 voxels was also used in this study.

## Results

### Physiological Data

Respiration and heart rates to subjects were monitored during the whole experimental session. The average values of individual respiration and heart rates were calculated and then compared before and after sleep deprivation using paired t-test. No differences were found regarding heart rate or respiratory rate between the RW and TSD conditions (Heart rate: RW, 68.42±7.26; TSD, 72.00±6.61; *t*(1,13) = −1.500; *p* = 0.161. Respiratory rate: RW, 19.01±2.42; TSD, 18.42±2.62; *t*(1,13) = 1.084; *p* = 0.300).

### Functional connectivity of the thalamus

**Figure 2:**
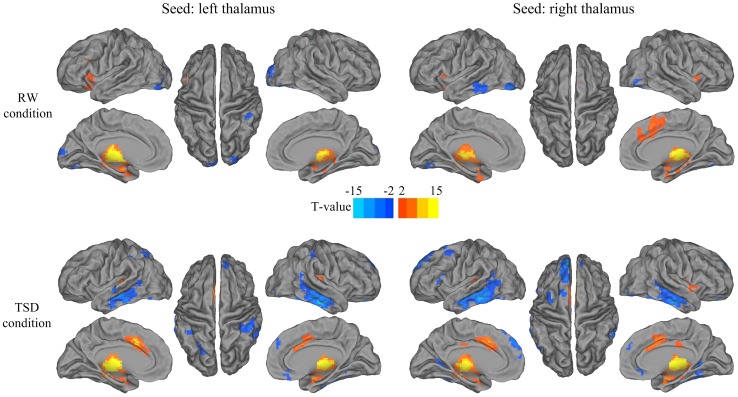
The whole-brain functional connectivity patterns of the left thalamus and right thalamus before and after the 36 h TSD. Brain regions with positive correlations are displayed in hot, while negative correlations are displayed in cold.

Fig. 2 shows the whole-brain functional connectivity patterns of the left thalamus and right thalamus. Significance level was set at *p*<0.001 uncorrected. Multiple comparisons were corrected at the cluster level (*p*<0.05, FDR corrected). We can see that the left thalamus and right thalamus were highly correlated with each other both in the RW and TSD conditions. However, after 36 h TSD, multiple temporal and prefrontal regions became negatively correlated with the thalamus.

### Decreased thalamus functional connectivity after TSD

**Figure 3:**
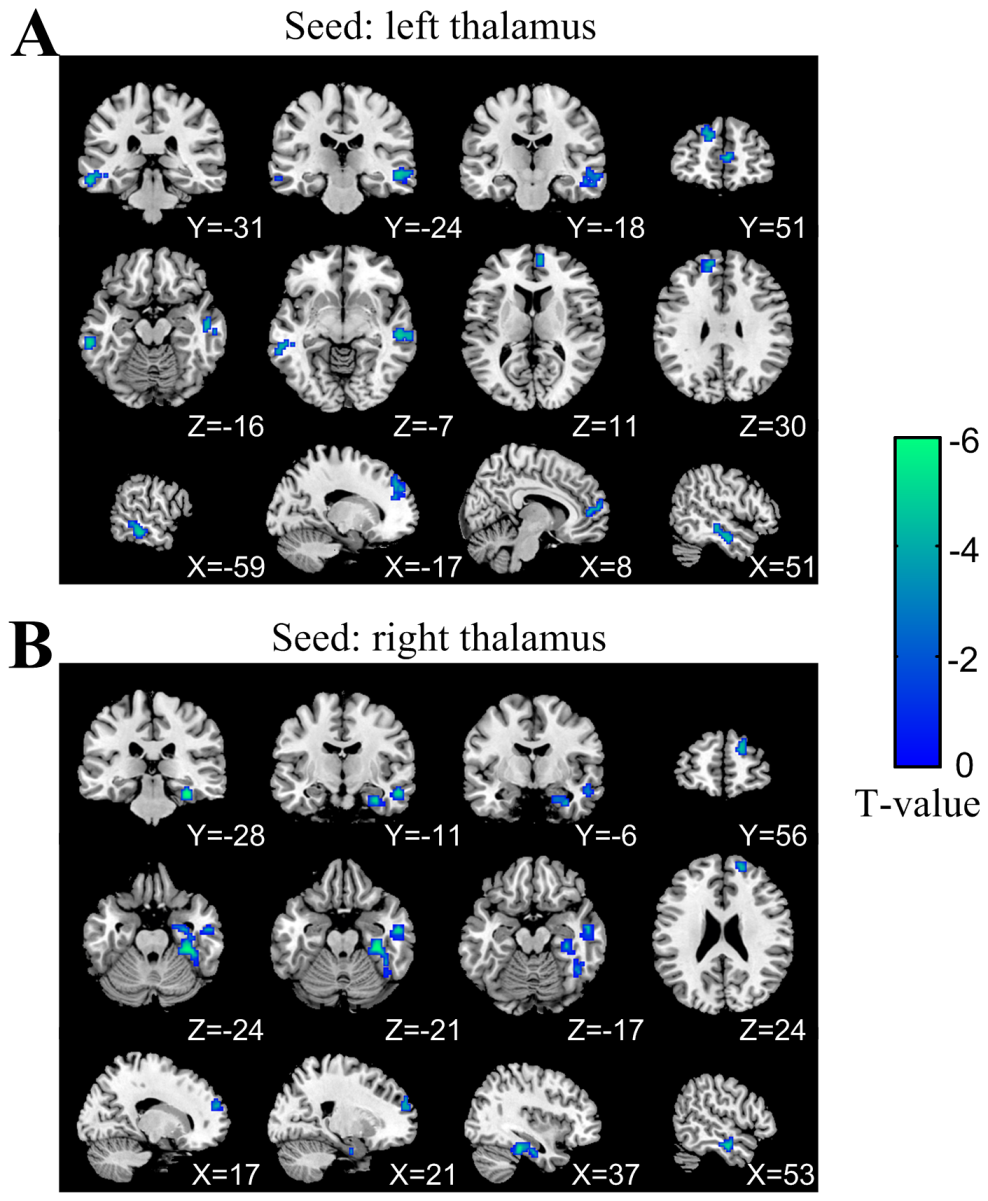
Brain areas that exhibited decreased functional connectivity with (A) the left thalamus and (B) right thalamus after 36 h TSD. In the transverse and coronal views, the left side of the images represents the left hemisphere of the brain.

Using the left thalamus and right thalamus as the seed regions, brain areas that exhibited significant TSD-induced functional connectivity changes are shown in Fig. 3 and Table 1. For the left thalamus, subjects showed decreased functional connectivity in the right medial frontal gyrus, bilateral middle temporal gyri and left superior frontal gyrus after 36 h TSD. For the right thalamus, subjects showed decreased functional connectivity in the right parahippocampal gyrus, right middle temporal gyrus and right superior frontal gyrus after 36 h TSD. There was no brain region that exhibited significantly increased functional connectivity with the left or right thalamus.

**Table 1:** Anatomical localization, cluster size, MNI coordinates and max T-values of TSD induced functional connectivity changes.

		MNI coordinates			
Anatomical area	Size	x	y	z	T-value
Seed: left thalamus (after TSD<before TSD)					
R medial frontal gyrus*	33	6	50	8	-5.9
L middle temporal gyrus**	57	−60	−31	−16	−5.4
R middle temporal gyrus**	87	54	−10	−22	−5.2
L superior frontal gyrus**	57	−12	50	29	−4.6
Seed: right thalamus (after TSD<before TSD)					
R parahippocampal gyrus**	118	33	−28	-22	−6.6
R middle temporal gyrus*	32	54	−10	−19	−5.5
R superior frontal gyrus*	30	18	56	23	−4.7

** *p*<0.001 at the voxel level (uncorrected) with *p*<0.05 at the cluster level (FDR corrected). * *p<*0.001 at the voxel level (uncorrected) with cluster size > 20 voxels. L: left; R: right.

**Figure 4:**
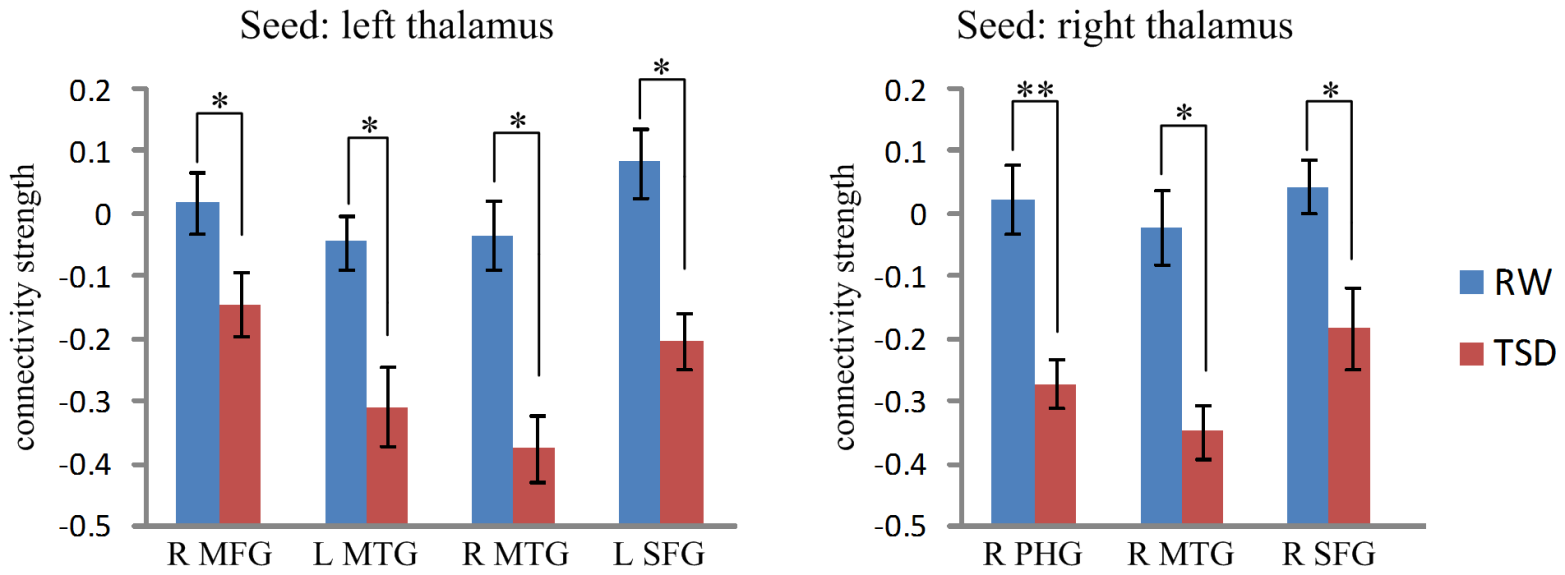
Correlation values between the thalamus and prefrontal and temporal ROIs in the RW and TSD conditions. MFG: medial frontal gyrus, MTG: middle temporal gyrus, SFG, superior temporal gyrus; PHG, parahippocampal gyrus. The error bars represent standard error of mean. The error bars represent standard error of mean. *: *p*<0.001, **: *p*<0.0001, paired *t*-test.

We further performed a region of interest (ROI) analysis to confirm our results. ROIs were defined as 6-mm-radius spheres centered on voxels which exhibited the largest absolute T-values in each of the significant clusters in Table 1. The mean functional connectivity strengths of these ROIs in the RW and TSD conditions are shown in Fig. 4. It can be seen that the connectivity strength became negative in all of these ROIs in the TSD condition. This uniform pattern of functional connectivity changes suggests that the thalamus may down-regulate cortical activity through synchronized oscillations after TSD.

## Discussion

In this paper, we examined the effects of TSD on thalamocortical functional connectivity during a resting state. The results showed that after sleep loss in the second night, the functional connectivity between the thalamus and the superior frontal gyrus, the temporal gyrus, the parahippocampal gyrus reduced in the resting brain when compared with that after normal sleep. Our results indicate that functional networks linking the cerebral cortex to the thalamus are abrupt after TSD. To the best of our knowledge, this is the first study to explore the patterns change of the thalamuocortical functional connectivity after TSD. Our study added new evidence for the decline hypothesis by investigating the thalamus-related connectivity with all brain regions. It suggests that TSD can cause disruptive changes of the thalamocortical functional connectivity, which may underwent the decline of the arousal level and information integration, and then influence the attention, memory and other brain functions.

We found that after 36 h TSD, multiple temporal and prefrontal regions became negatively correlated with the thalamus. Until now, there is no convergent conclusion about the underlying mechanisms of negative functional connectivity. Although some studies show that the negative correlation can be introduced by whole-brain signal regression [18], other studies demonstrate that it reflects active decoupling of different functional systems under task condition [19], [20]. Moreover, another study suggests that the negative functional connectivity may reflect the result of phase accumulation along the shortest path in brain functional networks [21]. Therefore, in this study, we speculated that anticorrelation in spontaneous BOLD activity operates to prevent different brain regions from communication with each other [22].

We found that the superior prefrontal cortex (PFC) appear to have declined connectivity to the left and right thalamus after TSD. The PFC has a unique executive attention role in actively maintaining assess to stimulus representations and goals in interference-rich contexts, and has been identified before as the first region to particularly sensitive to sleep deprivation [4]. Some higher cognitive deficits may result from altered functioning in prefrontal executive systems [1], [23]. Most importantly, the PFC is among the arousal attention network, a network consisting of functionally linked lateral thalamus, and other brain regions [24]. In an EEG study during quiet wakefulness, theta power is highest over frontal regions, and further increases was observed after sleep deprivation, indicating the enhanced mental fatigue after sleep loss [25]–[27]. Reported effects of TSD on glucose metabolism as measured by 18-FDG-PET as decreased metabolism both in absolute terms and after correction for the global decrease were found mainly in the prefrontal cortex, the parietal cortex and the thalamus [13]. Our results suggested that SD not only affects activity and metabolism in the prefrontal regions, but also caused the fragmentation of prefrontal-subcortical networks. Since these brain areas are vital to the arousal attention of the body, we can conclude the decreased connectivity between the thalamus and the prefrontal cortex regions may underwent the attention decline after long-time SD.

In this study, the decline of functional connectivity between the thalamus and the prefrontal cortex may add new evidence for the adaptation of the brain to TSD. The medial PFC is among the DMN, a network consisting of functionally linked PCC/precuneus, medial frontal and inferior parietal regions [28]–[30]. Connectivity of the DMN played an important role in human cognition, including the integration of cognitive and emotional processing, monitoring the world around us. Significantly, it has also been found that reduced connectivity of the DMN is associated with poor cognitive performance across a range of domains [31]. As we known from the previous studies that the DMN presented a reduced neuro-connection after SD [4], [17]. Individuals need to use more brain resources to maintain arousal levels and wakefulness after TSD, particularly with the declined FC of DMN and other brain network. Since the thalamus is a subcortical region that has been shown to play an intermediary role in cortico-cortical interactions [12], the declined functional connectivity reveals the decreased control of the cortex on subcortical areas. Thus, the reduced functional connectivity between the thalamus and the medial PFC after TSD may lead to insufficient allocation of cognitive resources to motor and attention systems and, in consequence, poor performance.

In the temporal lobe regions, we found that the middle temporal gyrus (MTG) showed disrupted functional connectivity to thalamus after TSD. This region is regarded as an important brain structure in the integration of memory, audiovisual association, and object-recognition functions [32], [33]. In resting state, the declined functional connectivity between the MTG and the thalamus may indicate the impaired integration of multimodal information after TSD. Besides, we observed that the parahippocampal gyrus showed decreased functional connectivity with the thalamus after TSD. Though the hippocampus is critical for memory, the surrounding medial temporal cortex such as the parahippocampal gyrus also plays an important role in establishing memories [34], [35]. Therefore, altered connectivity of the parahippocampal gyrus may be related to dysfunctions of memory formation and retrieve after TSD. Moreover, some previous studies have also reported that the parahippocampal gyrus is involved in complex emotional stimuli [36], so the decreased functional connectivity between the thalamus and the parahippocampal gyrus may also suggest the impairment of the human emotion functions. It is the emotion impacted on the attention arousal level or not need to be confirmed in future study. Then we concluded that the declined functional connectivity between the MTG, parahippocampal gyrus and the thalamus may reflect the disengagement of the conscious brain from the external world after TSD.

There are also several limitations in our study. First, the sample size is relatively small, thereby limiting the statistical power to detect group differences. Second, although subjects included in our analysis reported no sleep during the resting state scan, reproduction of the results here and functional connectivity reduction under EEG monitoring are needed to exclude any intrusion of sleep. Third, due to the limit of experimental conditions and the long time course for subjects to accomplish the experiment, women subjects were not recruited in this study. In the future, it is interesting to investigate gender differences of functional connectivity changes in sleep deprivation. Finally, sleep deprivation studies in larger groups may also target other resting state networks, and the emotion impacted on the attention arousal level or not need to be confirmed in future study.

## Conclusions

In summary, the current study used resting-state fMRI to examine how TSD impact on the thalamocortical functional connectivity network, and provide new evidence to the reduced integrity communication in thalamus-related network after TSD. Our findings may help to shed light on the neural mechanisms of the decline of arousal level and cognitive functions after TSD.
